# Glucose metabolism and its role in the maturation and migration of human CD1c^+^ dendritic cells following exposure to BCG

**DOI:** 10.3389/fcimb.2023.1113744

**Published:** 2023-07-05

**Authors:** Denise Triglia, Karl M. Gogan, Joseph Keane, Mary P. O’Sullivan

**Affiliations:** ^1^ TB Immunology Laboratory, Department of Clinical Medicine, Trinity Translational Medicine Institute, Trinity College Dublin, The University of Dublin, Dublin, Ireland; ^2^ Department of Respiratory Medicine, St James Hospital, Dublin, Ireland

**Keywords:** dendritic cell, immunometabolism, tuberculosis, glycolysis, *Mycobacterium tuberculosis*, *Mycobacterium bovis*, vaccines, Bacille Calmette-Guérin vaccine (BCG)

## Abstract

**Introduction:**

Tuberculosis (TB) still kills over 1 million people annually. The only approved vaccine, BCG, prevents disseminated disease in children but shows low efficacy at preventing pulmonary TB. Myeloid dendritic cells (mDCs) are promising targets for vaccines and immunotherapies to combat infectious diseases due to their essential role in linking innate and adaptive immune responses. DCs undergo metabolic reprogramming following exposure to TLR agonists, which is thought to be a prerequisite for a successful host response to infection. We hypothesized that metabolic rewiring also plays a vital role in the maturation and migration of DCs stimulated with BCG. Consequently, we investigated the role of glycolysis in the activation of primary human myeloid CD1c^+^ DCs in response to BCG.

**Methods/results:**

We show that CD1c^+^ mDC mature and acquire a more energetic phenotype upon challenge with BCG. Pharmacological inhibition of glycolysis with 2-deoxy-D-glucose (2-DG) decreased cytokine secretion and altered cell surface expression of both CD40 and CCR7 on BCG-challenged, compared to untreated, mDCs. Furthermore, inhibition of glycolysis had differential effects on infected and uninfected bystander mDCs in BCG-challenged cultures. For example, CCR7 expression was increased by 2-DG treatment following challenge with BCG and this increase in expression was seen only in BCG-infected mDCs. Moreover, although 2-DG treatment inhibited CCR7-mediated migration of bystander CD1C^+^ DCs in a transwell assay, migration of BCG-infected cells proceeded independently of glycolysis.

**Discussion:**

Our results provide the first evidence that glycolysis plays divergent roles in the maturation and migration of human CD1c^+^ mDC exposed to BCG, segregating with infection status. Further investigation of cellular metabolism in DC subsets will be required to determine whether glycolysis can be targeted to elicit better protective immunity against Mtb.

## 1 Introduction

Infection with *Mycobacterium tuberculosis* (Mtb), the causative agent of tuberculosis (TB), was the second leading cause of death by a single infectious agent in 2021 - killing 1.6 million people (WHO, 2021). New cases of multi-drug resistant and extensively-drug resistant TB are emerging every year. Consequently, the need for an effective vaccine is clear. Bacille Calmette-Guérin (BCG), a live attenuated strain of *M. bovis*, is the only vaccine licensed to prevent TB disease. Although BCG vaccination does not prevent primary infection with Mtb (Moliva et al., 2017), it is effective at preventing TB meningitis and extra pulmonary disseminated TB in children (Rodrigues et al., 1993; Trunz et al., 2006). BCG also affords limited protection against non-tuberculous mycobacterial infections such as Buruli ulcer and leprosy (Setia et al., 2006; Zimmermann et al., 2018). However, its efficacy at protecting against adult pulmonary TB varies from zero to 80% in different populations (Rodrigues et al., 1993).

Despite its shortcomings, BCG vaccination of high-risk individuals is still a key component of the WHO’s End TB Strategy (WHO, 2021) and BCG remains the benchmark against which candidate vaccines are measured. BCG is one of the most widely used vaccines globally and is administered *via* the intradermal route to neonates in countries with a high burden of TB – with approximately 100 million babies vaccinated each year (WHO, 2008). BCG is also an integral part of several new vaccination strategies tested in clinical trials, including recombinant strains with improved immunogenicity (Loxton et al., 2017; Nieuwenhuizen and Kaufmann, 2018), subunit vaccines designed to boost the immune response elicited by BCG (Nemes et al., 2018; Van Der Meeren et al., 2018) and revaccination of adolescents (Nemes et al., 2018). BCG is also used as a first line immunotherapy for early stage bladder cancer (Lamm, 1985). Vaccination with BCG also confers heterologous protection against a variety of infectious diseases unrelated to TB (Kleinnijenhuis et al., 2014; Higgins et al., 2016) (Giamarellos-Bourboulis et al., 2020) as well as showing therapeutic potential in protection against certain autoimmune diseases (Ristori et al., 2014; Kuhtreiber et al., 2018).

Trafficking of DCs infected with live bacilli to the draining lymph node (LN) is essential for the generation of an antigen-specific immune response following infection with mycobacteria (Marino et al., 2004; Reiley et al., 2008; Wolf et al., 2008; Samstein et al., 2013; Bollampalli et al., 2015) although virulent strains of mycobacteria can also impair the ability of infected DCs to migrate (Rajashree et al., 2008; Roberts and Robinson, 2014; Lai et al., 2018). Pulmonary delivery of Mtb antigen-primed DCs into naïve (Gonzalez-Juarrero et al., 2002; McShane et al., 2002) and vaccinated mice (Griffiths et al., 2016) has been shown to accelerate T-cell responses to Mtb infection. In addition, CD11c^+^ DCs have been successfully targeted with mycobacterial antigens conjugated to a DC-Sign antibody to produce protective T cell responses (Velasquez et al., 2018). Myeloid or conventional (m/cDCs) are one of the major cell populations infected with Mtb in the lung and draining lymph nodes (LN) in mouse models of TB (Wolf et al., 2007) and in humans comprise of three subsets: cDC1, CD1c^+^ cDC2 and the recently identified inflammatory cDC3 which also express CD1c in addition to CD163 and CD14 (Segura, 2022). The murine counterpart of human CD1c^+^ mDCs - CD11b^+^ cDCs - initiate Th1 T cell immunity during pulmonary Mtb infection (Lai et al., 2018). Moreover, migratory epidermal CD11b^hi^ cDCs transport BCG from the skin to draining LN to prime CD4^+^ T cells following BCG inoculation (Bollampalli et al., 2015; Krmeska et al., 2022). Human blood-derived CD1c^+^ mDCs are susceptible to infection with BCG, up-regulating CD40, CCR7, HLA-DR, CD86 and other maturation markers, as well as producing TNF-α and IL-6 (Lozza et al., 2014). However, the intracellular processes that enable primary human CD1c^+^ mDCs to perform these immune functions in response to bacterial infection are unknown.

Interactions between innate immune cells and microorganisms can result in changes in host cell metabolism that shape the subsequent immune response. A shift from oxidative phosphorylation (OXPHOS) to aerobic glycolysis is required for maturation of murine bone marrow derived DCs (BMDCs) stimulated with LPS and eventually results in an almost complete abrogation of mitochondrial metabolism (Krawczyk et al., 2010; Everts et al., 2014). Human CD1c^+^ mDCs stimulated with single-stranded RNA, a TLR7/8 agonist, also undergo similar metabolic reprogramming to support their maturation (Basit et al., 2018). Our group previously showed that a metabolic switch to aerobic glycolysis plays an important role in the control of Mtb infection by macrophages (Gleeson et al., 2016). Here, we investigated the metabolic requirements of human CD1c^+^ mDCs in response to the BCG vaccine. Upon challenge with BCG, CD1c^+^ mDCs exhibited increased rates of OXPHOS and glycolysis. Inhibition of glycolysis with 2-Deoxy-D-Glucose (2-DG) decreased cytokine secretion and decreased the cell surface expression of CD40 in BCG-challenged cells. Unexpectedly, we found that inhibition of glycolysis had distinct effects on bystander and directly infected DCs, being required for optimal cell surface expression of CD40 and for CCR7-mediated migration of bystander cells, while both these functions were independent of glycolysis in BCG-infected cells. Our results suggest that the glycolytic pathway plays an important but nuanced role in the function of CD1c^+^ mDCs in the response to mycobacterial infection. A better understanding of the metabolic response of DCs to BCG vaccination may aid the development of a vaccine that stimulates more effective protection against Mtb.

## 2 Materials and methods

### 2.1 Mycobacteria

Bacillus Calmette-Guerin (BCG) expressing GFP (green fluorescent protein) was a gift from V. Deretic (University of New Mexico, Albuquerque, NM). Stocks were propagated in Middlebrook 7H9 (Difco; Becton Dickinson) containing 10μg/ml Kanamycin (Sigma-Aldrich). Aliquots were stored at -80°C, thawed and grown to log phase in Middlebrook 7H9 medium before use.

### 2.2 CD1c^+^ mDC isolation

Human CD1c^+^ myeloid DCs (mDCs) were isolated from blood samples obtained from patients with no known infections attending the Haemochromatosis Clinic in St. James’s Hospital after informed consent, as approved by the St. James’s Hospital and Tallaght University Hospital Joint Research Ethics Committee. First, PBMCs were separated by density gradient centrifugation on Lymphoprep™ (Axis-Shield). Cells were re-suspended in DC isolation buffer (2mM EDTA, 0.05% BSA in PBS) and DCs were enriched from PBMCs by negative magnetic separation using the EasySep™ Human Pan-DC Pre-Enrichment Kit following the manufacturer’s instructions (STEMCELL Technologies). The negative fraction containing DCs was then stained in DC isolation buffer with antibodies against lineage markers (Lin) (CD3, CD14, CD16, CD19, CD20, CD56), HLA-DR, CD1c and CD304 for 20min on ice in the dark. CD1c^+^ mDCs were further purified by cell sorting according to the following staining: Lin^-^HLA-DR^+^CD304^-^CD1c^+^.

### 2.3 CD1c+ mDC culture and infection

CD1c^+^ mDCs were cultured in RPMI 1640 Medium (Lonza) containing 5% Human Serum (Sigma-Aldrich) without antibiotics and challenged with BCG on the same day of isolation. Unless otherwise stated, cells were seeded at a density of 7.5 x 10^5^ cells/well in 200μl and infected for 20h with BCG at a ratio of 10 bacilli:cell. Alternatively, mDCs were stimulated with a combination of the TLR ligands Resiquimod (R-848, 5μg/ml, Enzo Life Sciences) and Polyinosinic-Polycytidylic acid (poly(I:C), 20μg/ml, Sigma-Aldrich) for the same period. Where indicated CD1c^+^ mDCs were treated for 1h prior to, and for the duration of infection with 1mM 2-Deoxyglucose (2-DG, Sigma-Aldrich).

For analysis by confocal microscopy CD1c^+^ mDCs were challenged with GFP-BCG as outlined above, washed and fixed with 2% paraformaldehyde and then mounted onto Polysine slides (ThermoFisher Scientific). Cells were imaged with a 63X magnification oil immersion objective on a Leica SP8 confocal microscope using LAS X software (Leica).

### 2.4 Flow cytometry

Flow cytometry analysis (CyAn ADP, Beckman Coulter) and cell sorting (MoFlo XDP, Beckman Coulter) were performed with the following antibodies (anti-human): Lineage Cocktail-FITC (CD3, CD14, CD16, CD19, CD20, CD56), HLA-DR-BrilliantViolet 421 (L243), CD304-PE (12C2), CD1c-APC (V T-CD01.18), CD40-APC/Cy7 (5C3), CCR7- PerCP/Cy5.5 (G043H7), Zombie Red (or Zombie Aqua) Fixable Viability Dye and anti-mouse IgG1-PE (RMG1-1) (all from Biolegend); CD83-APC and CD86-PE (BD Pharmingen). CD1c^+^ mDCs were harvested following BCG infection or TLR stimulation and stained for 20 minutes on ice in the dark in DC isolation buffer. Cells were then washed and fixed in 2% paraformaldehyde when necessary. CD1c^+^ mDCs were gated on forward scatter and side scatter to exclude cell clumps and debris. Dead cells were excluded from analysis according to Zombie Red staining (or with Zombie Aqua when cells were stained with Texas red conjugated to Dextran). Unstained CD1c^+^ mDCs, BCG-GFP and FMO controls were used to determine negative populations and to adjust gates appropriately. GLUT1 (Metafora Biosystems) staining was performed according to the manufacturer’s instructions. Mean fluorescence intensity (MFI) and the percentage of positive cells was determined for CD83, CD86, CD40 and CCR7. Analysis was performed using FlowJo® (Tree Star, Inc).

### 2.5 Dextran uptake assay

Peripheral venous blood samples were obtained from healthy adult donors following informed consent. PBMCs were separated by density gradient centrifugation on Lymphoprep™ and cells were washed twice with Ca/Mg-free PBS. PBMCs were resuspended in RPMI supplemented with 2% human serum and 1mg/ml dextran conjugated to Texas red (ThermoFisher) (Wang et al., 2009). Cells were incubated for 24 h at 4°C (as a negative control) or 37°C, washed, stained with cell surface markers, and analysed by flow cytometry. Dextran uptake was measured in **l**ive CDC1^+^ mDCs which were defined as Zombie Aqua^-^ Lin^-^ HLA-DR^+^ CD11c^+^CD1c^+^ and CD304^-^ cells.

### 2.6 Cytokine measurements

Cytokine concentration was assessed in cell-free supernatants at 20h post-infection using Meso Scale Discovery Proinflammatory Panel 1 (human) (MSD, Gaithersburg, MD) for the detection of IFN-γ, IL-1β, IL-2, IL-4, IL-5, IL-8, IL-10, IL-12p70, IL-13, and tumor necrosis factor (TNF)-α. Assay was performed according to the manufacturer’s instructions and data analyzed using Discovery Workbench 3.0 software (MSD).

### 2.7 Migration assay

CD1c^+^ mDCs migration assay towards CCL19 was performed using 24-well Transwell plates containing 5μm pore polycarbonate membrane inserts (Corning Life Sciences). A total of 1x10^5^ CD1c^+^ mDCs were challenged with BCG, with or without 2-DG (1mM), for 20h. Following infection, the cells were harvested, counted and suspended in 200µl of culture medium and placed on the upper side of the membrane. A portion of the cells (“input”) was kept aside for analysis as detailed below. The remaining mDCs were allowed to migrate to the lower chamber containing 750µl of complete RMPI medium containing CCL19 (50ng/ml, Bio-Techne) for 3h at 37°C. Input and transmigrated cells were stained with 10μg/ml cell-permeable Hoechst 33342 (Thermo Scientific) and counted using the Cytell Cell Imaging System (GE Life Sciences). Results were expressed as the percentage of cells migrating to the lower chamber in relation to the initial cell number added to the upper chamber (input). Input and migrating cells, challenged with BCG with or without 2-DG treatment, were recovered, fixed with 2% paraformaldehyde and then mounted onto Polysine slides (ThermoFisher Scientific). 200 cells in each sample were counted using an Olympus IX51 fluorescent microscope with a 100X oil objective. Infected cells were detected using the FITC filter to visualize GFP and nuclei were visualized using the DAPI filter to count all of the cells. Changes in migration of bystander (GFP^-^) and directly infected (GFP^+^) cells were calculated as the ratio of the number of migrated cells (GFP^-^ or GFP^+^) to the original numbers of GFP^-^ or GFP^+^ cells added to the top of the transwell.

### 2.8 Metabolic assays

Real-time analysis of extracellular acidification rate (ECAR) and oxygen consumption rate (OCR) was determined in mDCs using an XFe24 Extracellular Flux Analyzer (Seahorse Biosciences). Briefly, 2x10^5^ CD1c^+^ mDCs were seeded onto Seahorse XF24 Cell Culture Microplates pre-coated with CellTak™ (1µg/well, Corning) and challenged with rifampacin-killed BCG-GFP at a ratio of 50 bacilli:cell for 20h in complete RPMI. CD1c^+^ mDCs were then carefully washed with Seahorse Media supplemented with 2mM L-glutamine and 10mM glucose. The Agilent Seahorse XF Cell Mito Stress Test Kit was used to assess mitochondrial function. The optimal concentrations of oligomycin and FCCP were determined in an initial pilot study. Measurement of baseline ECAR and OCR was carried out followed by successive injections of the following reagents to obtain the indicated concentrations: oligomycin (1 μM), FCCP (2 μM) and finally rotenone (0.5 μM) + antimycin A (0.5 μM). Glycolytic function was assessed in the cells using the Seahorse XF Glycolysis Stress Test Kit according to the manufacturer’s instructions. Cells were challenged with BCG-GFP as above and CD1c^+^ mDCs were then carefully washed with Seahorse Media supplemented with 2mM L-glutamine and incubated in a non-CO_2_ incubator for 1 hour prior to the run. Baseline ECAR and OCR were measured before and after injection of glucose (10mM) to determine basal rates of ECAR and OCR followed by injections of oligomycin (1 μM) and finally 2-DG (50mM). Results were normalized by cell number in each well which was determined by staining the cells with 10μg/ml Hoechst 33342 followed by analysis using the Cytell Cell Imager as described above.

L-Lactate concentration was measured in the cell supernatants at 20h p.i. with BCG by colorimetric assay in 96-well plates according to the manufacturer’s instructions (Sigma-Aldrich). The absorbance of samples and standards was read at 570 nm using an Epoch Microplate Spectrophotometer with Gen5 Data Analysis software (BioTek Instruments).

### 2.9 Statistical analysis

Statistical analysis was performed using Graph Pad Prism 9 Software (San Diego, CA, USA). Results are expressed as mean ± SEM. Data was considered non-parametric as per normality test performed on the data sets. Tests used were unpaired/paired two-tailed t test or Wilcoxon-ranked sum test as appropriate to compare two groups and Repeated Measures One-Way Analysis of Variance (ANOVA) to compare more than two sets of data followed by the recommended post tests. Repeated Measures or mixed model Two-Way ANOVA was used to compare more than two sets of paired grouped data followed by the recommended post tests. A *p* value < 0.05 was considered statistically significant.

## 3 Results

### 3.1 Challenge of CD1c^+^ mDCs with BCG results in up-regulation of surface markers and cytokine secretion

**Figure 1:**
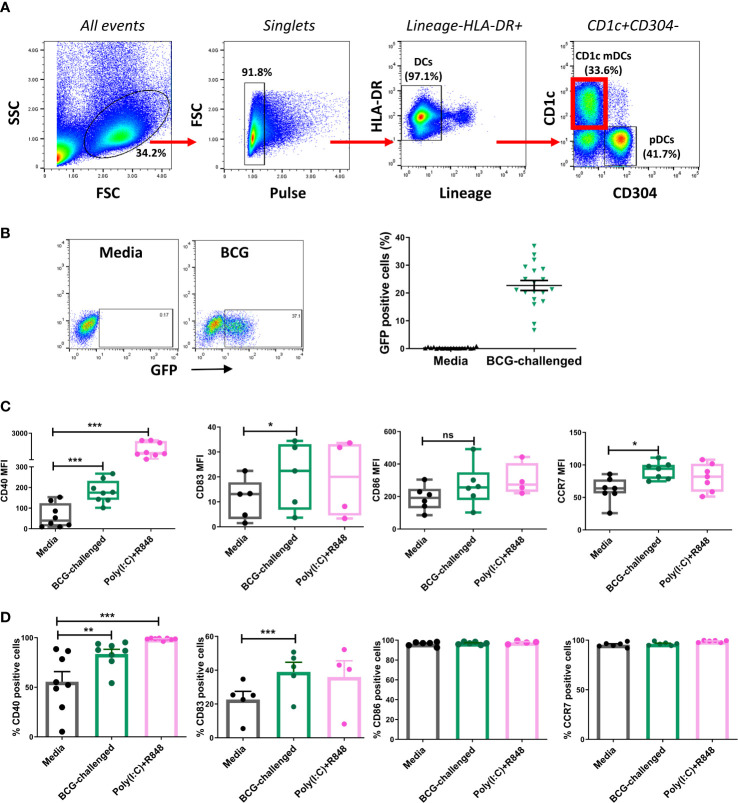
CD1c^+^ myeloid DCs respond to challenge with BCG through phagocytosis and up-regulation of maturation markers. **(A)** Gating strategy used for sorting CD1c^+^ mDCs. Dendritic cells were enriched from PBMCs by application of Pan-DC Pre-Enrichment Kit and purified by cell sorting. Lineage marker consisted of a cocktail of antibodies against CD3, CD14, CD16, CD19, CD20 and CD56 surface markers. Dot plots show one representative donor. **(B)** Percentage of infected (BCG-GFP^+^) CD1c^+^ mDCs at 20h post infection with MOI 10. Dot plots show one representative donor and graph shows individual and mean value ( ± SEM) from 19 different donors. **(C)** Surface marker levels for CD40 (n=8), CD83 (n=4-5) CD86 (n=4-5), CCR7 (n=7) shown as mean fluorescence intensity (MFI) in CD1c^+^ mDCs challenged with BCG (MOI 10) or combination of the TLR ligands poly(I:C) (20μg/ml) + R-848 (5μg/ml) compared to control (media). Box plots extend from the 25-75% interquartile range, the horizontal bar depicts the median and whiskers indicate the minimum to maximum values. Each dot represents the result from an individual donor. **(D)** Surface marker levels shown as percentage of positive cells. Each dot represents the result from an individual donor, bars show mean and SEM. Statistical significance was determined using a repeated measures one way analysis of variance (or mixed effects model where appropriate) with Dunnett’s multiple comparisons test comparing BCG and poly(I:C)+R-848 to the media control. * p< 0.05, **p<0.01 and ***p<0.001, ns, not significant.

Myeloid dendritic cells are very scarce, comprising of between 0.3% - 0.9% of all peripheral blood mononuclear cells. These cells have a short lifespan *in vitro* (Nopora and Brocker, 2002; Hou and Van Parijs, 2004) and can become activated upon culturing (Dzionek et al., 2000), facts that restrict the use of these cells *in vitro*. We first attempted to isolate CD1c^+^ mDCs from the buffy coats provided by the Irish Blood Transfusion Service facility in St James Hospital Dublin, Ireland. Following isolation of PBMCs from blood and enrichment of the DC population by magnetic beads, CD1c^+^ mDCs were sorted according to expression of the surface markers Lineage^-^, HLA-DR^+^, CD1c^+^ and CD304^-^ (**Figure 1A**). However, buffy coats are available for processing only 48 hours after they are drawn, which could explain the low yield of DCs isolated from these buffy coats: the number of CD1c^+^ mDCs obtained by this methodology ranged from 4.2x10^3^ to 0.7x10^6^ cells (mean = 0.30x10^6^, SD ± 0.28x10^6^) per buffy coat. For this reason, we isolated CD1c^+^ mDCs from fresh blood collected from patients with Hereditary Hemochromatosis (HH). The number of CD1c^+^ mDCs obtained from one unit (approximately 500ml) of freshly drawn blood was significantly higher and ranged from 4x10^5^ to 1x10^6^ cells with purity of 94.03% (SD ± 4.15%, n= 62). Our preliminary analysis showed no significant differences regarding expression of surface markers and endocytic capacity (assessed by Dextran-internalization assay) between mDCs from HH patients and healthy controls (**Supplementary Figure 1**).

In keeping with a previously published report (Dzionek et al., 2000) confocal microscopy of the sorted CD1c^+^ mDCs revealed that they were medium-sized cells with hyperlobulated nuclei (**Supplementary Figure 2A**). Purified CD1c^+^ mDCs were challenged with a GFP-tagged strain of BCG, at a ratio of 10:1 mycobacteria:cell or stimulated with a mixture of the TLR ligands poly(I:C) and R-848 for 20h. Analysis by flow cytometry showed that an average of 22.7% (SD ± 7.8) of the mDCs had successfully phagocytosed bacilli (**Figure 1B**). Z-stack imaging of BCG-challenged mDCs (n=5 individual donors) indicated that the bacteria were located within the infected cells rather than adhering to the surface (**Supplementary Figure 2B**).

The maturation status of the CD1c^+^ mDCs following infection was performed by assessing expression of maturation markers on the cell surface and cytokine secretion on the cell supernatants. Challenge with BCG or stimulation with poly(I:C)/R-848 resulted in up-regulation of the surface markers CD83, CD40 and CCR7. Expression of CD86 trended towards an increase but did not reach statistical significance with either stimulus (**Figure 1C, D**).

### 3.2 Challenge with BCG induces changes in the cell metabolism of humans CD1c^+^ mDCs

**Figure 2:**
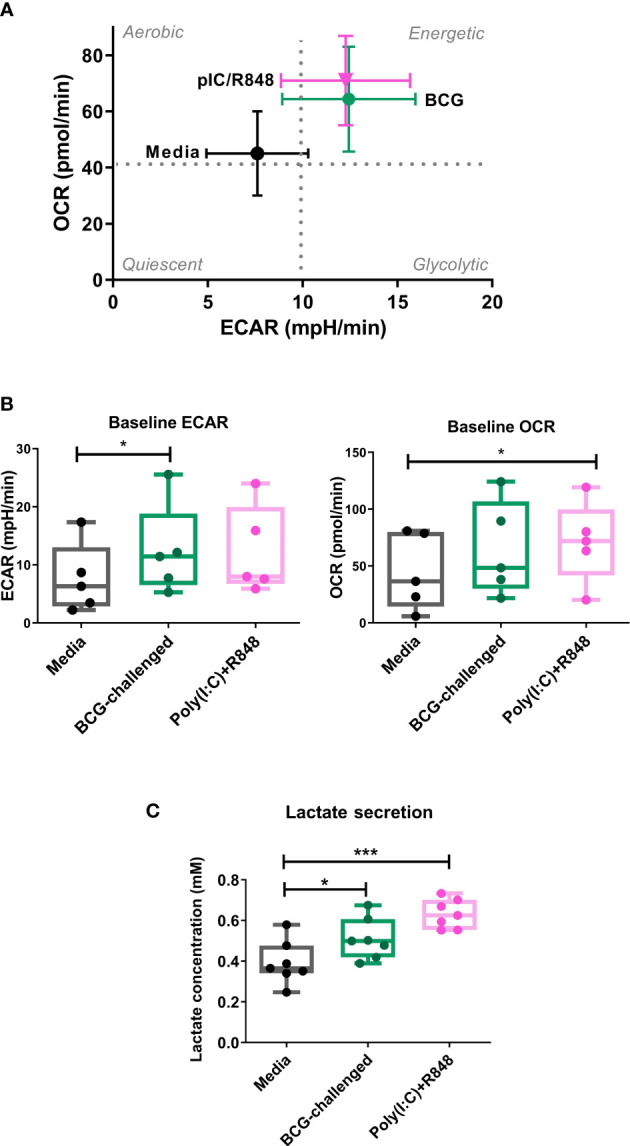
Metabolic profile of CD1c^+^ mDCs in response to BCG. **(A)** XF Cell Energy Phenotype of uninfected and BCG-challenged CD1c^+^ mDCs at 20h p.i. Plot shows average of baseline readings ( ± SEM) for 5 donors of oxygen consumption rates (OCR) and extracellular acidification rates (ECAR) obtained with the Seahorse Extracellular Flux Analyzer. Box plots extend from the 25-75% interquartile range, the horizontal bar depicts the median and whiskers indicate the minimum to maximum values. Superimposed dots represent the results from five individual donors. **(B)** Lactate secreted in the cell supernatants measured at 20h p.i with BCG challenge or stimulation with TLR ligands. Box plots extend from the 25-75% interquartile range, the horizontal bar depicts the median and whiskers indicate the minimum to maximum values. Superimposed dots represent the results from seven different donors. Statistical significance was determined using a repeated measures one way analysis of variance with Dunnet’s multiple comparisons test. * *p*< 0.05, ***p<0.001.

Increasing evidence has reinforced the importance of metabolic reprogramming in licensing immune cells to perform their effector functions. TLR stimulation of murine DCs boosts their rate of aerobic glycolysis, which is necessary to meet increased biosynthetic demand during maturation (Krawczyk et al., 2010; Everts et al., 2014; Thwe and Amiel, 2018). A shift towards glycolysis is also necessary for human macrophages to control infection with Mtb (Gleeson et al., 2016). Therefore, we hypothesized that human CD1c^+^ mDCs would also undergo metabolic reprogramming upon challenge with BCG and decided to characterize the energetic profile of these cells. We first analyzed the baseline extracellular acidification rate (ECAR) and oxygen consumption rate (OCR) of immature and BCG-challenged CD1c^+^ mDCs at 20h p.i using a Seahorse metabolic analyzer in the presence of glucose (**Figure 2A**). We observed that BCG and poly(I:C)/R848 modestly increased the rates of glycolysis (ECAR measurement) and mitochondrial respiration (OCR measurement) in CD1c^+^ mDCs, resulting in a more energetic phenotype (**Figure 2B**). An examination of mitochondrial function using the Mito Stress Test (**Supplementary Figure 3**) revealed a trend towards increased basal OCR in BCG-challenged cells and maximal respiration was significantly higher with BCG challenge compared to the control, indicating that they have an increased capacity to oxidise mitochondrial fuels when stressed. In addition, non-mitochondrial OCR was significantly decreased in BCG-challenged mDCs compared to control cells. The increase in baseline OCR observed in poly(I:C)/R848-treated cells was primarily due to an increase in non-mitochondrial OCR, possibly due to increased cytosolic ROS production.

Lactate is an end product of glycolysis, and its production is increased as a result of LPS-mediated enhancement of glycolytic flux in dendritic cells (Jantsch et al., 2008; Everts et al., 2012). To confirm an increase in glycolysis in our model, in parallel we measured the levels of lactate secreted in the cell supernatants. Lactate production was significantly increased in BCG-challenged CD1c^+^ mDCs in comparison to uninfected control cells in response to both BCG and the TLR ligands poly(I:C)/R-848 (**Figure 2C**). Increased glycolytic flux can also be accompanied by increases in the expression of glucose transporters on the cell surface. We measured the expression of Glucose Transporter 1 (GLUT1) on CD1c^+^ mDCs by flow cytometry but no significant differences were observed between the different groups (**Supplementary Figure 4**). Taken together, these results suggest that challenge with BCG results in changes in cell metabolism of CD1c^+^ mDCs, inducing a more energetic phenotype and increased lactate production but without a concomitant increase in GLUT1 cell surface expression.

### 3.3 Challenge with BCG boosts the glycolytic function of CD1c^+^ mDCs

**Figure 3:**
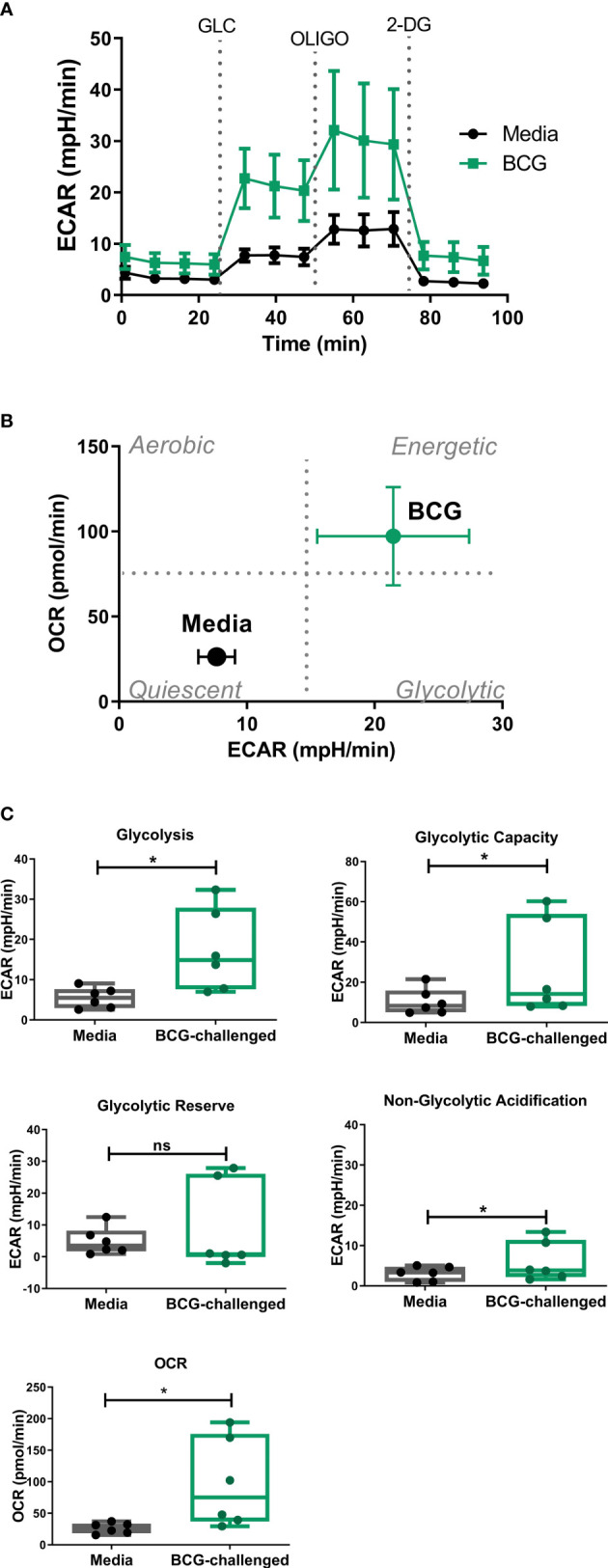
Analysis of glycolytic function in CD1c^+^ mDCs following challenge with BCG. **(A)** ECAR readings (mean ± SEM) for six independent donors) of CD1c^+^ mDCs subjected to the Glycolysis Stress test which were initially cultured in glucose-free medium and ECAR was measured following glucose (Glu), oligomycin (Oligo), and 2-Deoxy-D-glucose (2-DG) injections in control and BCG-challenged CD1c^+^ mDCs at 20h p.i. **(B)** XF Cell Energy Phenotype of uninfected and BCG-challenged CD1c^+^ mDCs (n=6) at 20h p.i. ECAR changes as determined by Seahorse metabolic profiling. Plot shows average of basal readings (± SEM) for oxygen consumption rates (OCR) and extracellular acidification rates (ECAR) after the addition of 10mM glucose obtained with the Seahorse Extracellular Flux Analyzer. **(C)** Box plots depict Glycolysis rates (ECAR after glucose addition subtracted from baseline ECAR before the first injection), Glycolytic Capacity (ECAR after oligomycin infection subtracted from basal ECAR) and Glycolytic Reserve (glycolytic capacity minus basal glycolysis) calculated from the ECAR curve. Box plots extend from the 25-75% interquartile range, the horizontal bar depicts the median and whiskers indicate the minimum to maximum values. Superimposed dots represent the results from six independent donors. Statistical significance was determined using paired t-tests. * *p*< 0.05, ns, not significant.

As the glycolytic pathway has been shown to have an important role in survival and function of DCs and basal rates of glycolysis were significantly increased following exposure to BCG, we examined glycolytic function of CD1c^+^ mDCs in response to BCG infection in more detail. To specifically interrogate extracellular glucose-driven metabolism we performed a glycolysis stress test of BCG-challenged CD1c^+^ mDCs in comparison to naive cells. Cells were exposed to BCG as described before and the culture medium was replaced with glucose-free medium for 1 hour prior to the assay. Then, ECAR was measured at baseline levels and again after re-introduction of glucose to determine the basal glycolytic rate. Next, oligomycin was added to the cells to block mitochondrial respiration and promote maximal levels of glycolysis and, finally, glycolysis was blocked by addition of 2-DG to the cells (**Figure 3A**). With this methodology it was possible to determine the basal glycolytic rate (difference between ECAR before and after glucose addition), glycolytic capacity (difference between ECAR following injection with oligomycin and basal readings) and glycolytic reserve (difference in ECAR between addition of glucose and oligomycin) of the cells. Both uninfected and BCG-infected CD1c^+^ mDCs increased ECAR following addition of glucose to the cells (**Figure 3A**) but the basal glycolytic rate was significantly higher for BCG-infected cells (**Figure 3B**). In addition, OCR was increased in BCG-challenged cultures following the addition of glucose. (**Figures 3B, C**). BCG-infected cells also showed enhanced glycolytic capacity when compared to uninfected cells. However, we observed that infection with BCG did not alter the glycolytic reserve of the CD1c^+^ mDCs. These data indicate that BCG challenge increases the glycolytic rate and boosts the capacity of CD1c^+^ mDCs to use glucose to produce ATP *via* glycolysis when stressed. Non-glycolytic acidification was also significantly increased in BCG-challenged cultures compared to control (**Figure 3C**).

### 3.4 Inhibition of glycolysis decreases cytokine secretion of BCG-challenged CD1c^+^ mDCs

**Figure 4:**
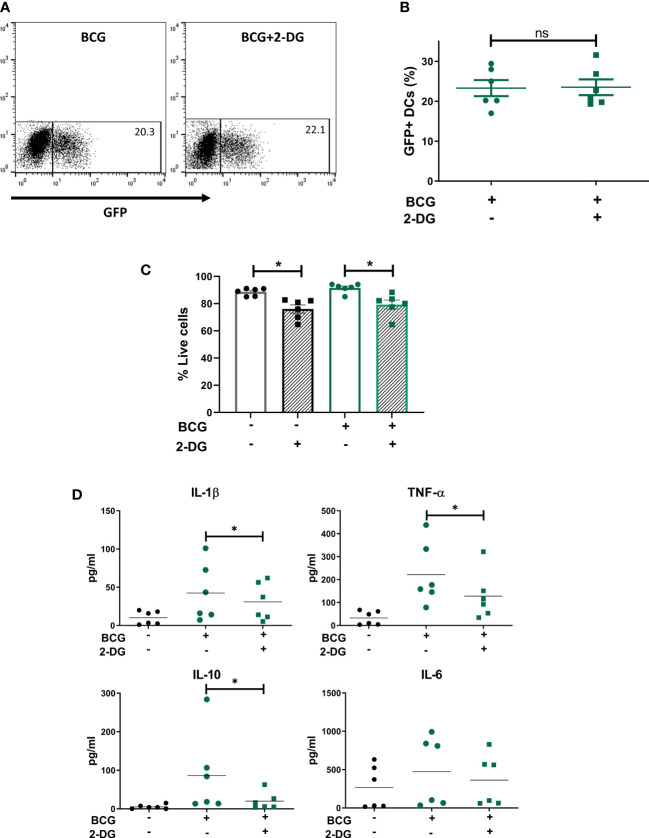
Inhibition of glycolysis decreases secretion of cytokines but does not affect phagocytosis of BCG by CD1c^+^ mDCs. **(A)** Representative dot plots from one donor showing percentage of GFP^+^ untreated or 2-DG treated CD1c^+^ mDCs and graph **(B)** showing percentage of BCG^+^ cells (mean value ± SEM) of six independent donors at 20h upon infection with BCG in cells pre-treated or not with 2-DG (1mM). **(C)** CD1c^+^ mDCs were treated or not with 1mM 2-DG and then challenged with BCG for 20h. Viability (% live cells) was then determined by staining the cells with Zombie red dye followed by analysis by flow cytometry. Cells that were negative for Zombie red were considered viable. Bar charts show mean (± SEM) and superimposed dots represent the results from six independent donors. Statistical significance was determined by two-way repeated measures analysis of variance followed by Šídák’s multiple comparison test. **(D)** Cytokine production was measured in the cell supernatants at 20h p.i. Graphs show values (± SEM) from six independent donors. Statistical significance was determined using a Wilcoxon signed-rank test, * *p*< 0.05, ns, not significant.

To understand the role of the glycolytic pathway in CD1c^+^ mDC function in response to BCG, we pretreated the cells with the glycolysis inhibitor 2-DG prior to infection. We first investigated if inhibition of glycolysis affected the phagocytic capacity of the CD1c^+^ mDCs. However, pre-treatment with 2-DG had no impact on the level of BCG infection when compared to untreated control cells (**Figures 4A, B**) although it had a small but significant effect on viability of BCG challenged DCs (**Figure 4C**). We then assessed the effect of 2-DG on cytokine production by the CD1c^+^ mDCs following challenge with mycobacteria. Exposure to BCG induced secretion of the cytokines IL-1β, IL-6, TNF-α and IL-10 (**Figure 4D**). Production of IL-12p70, IFN-γ and low levels of IL-4 was only detected after TLR stimulation but not in BCG-challenged cells (data not shown). These results are consistent with previous findings (Lozza et al., 2014) and suggest no functional abnormalities in response to infection with BCG in the CD1c^+^ mDCs used in this study.

**Figure 5:**
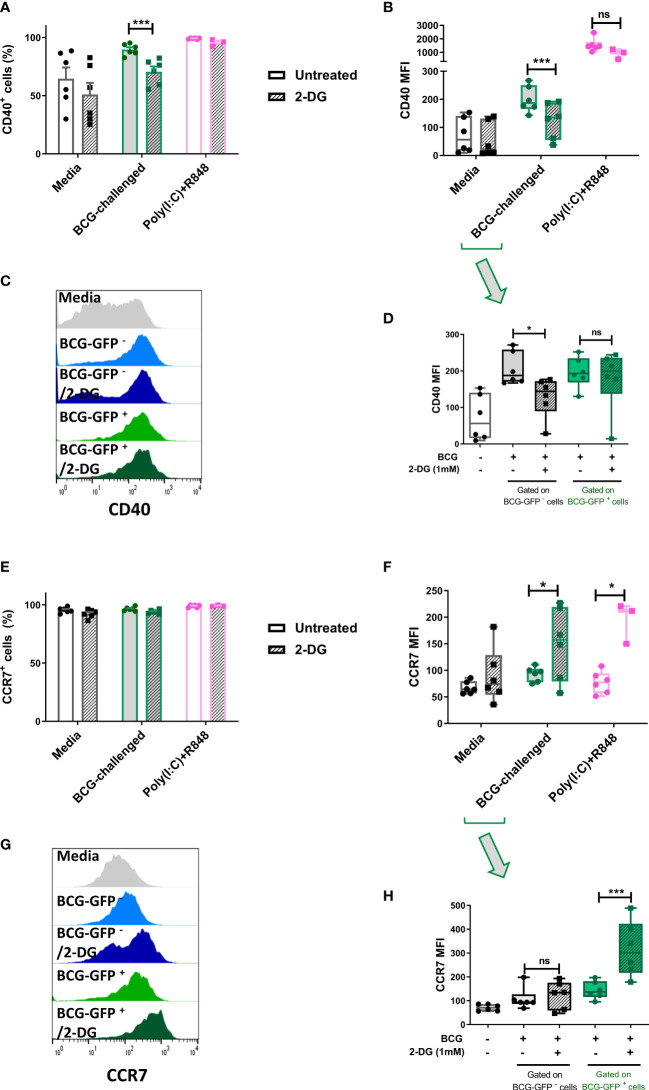
Inhibition of glycolysis has differential effects on cell surface expression of CD40 and CCR7 of bystander and BCG-infected CD1c^+^ mDCs. Surface levels of CD40 and CCR7 were analyzed by flow cytometry at 20h p.i. with BCG in CD1c^+^ mDCs pre-treated or not with 2-DG (1mM). **(A)** Percentage of CD40 positive and **(E)** CCR7 positive and **(B)** MFI of CD40 and **(F)** CCR7 positive live CD1c^+^ mDCs with and without preincubation with 2DG when gating strategy comprised all live cells. Graphs show mean fluorescence intensity (MFI). **(D)** CD40 and **(H)** CCR7 levels resulting from analysis in which the BCG-challenged CD1c^+^ mDCs that had phagocytosed BCG (BCG-GFP^+^) were gated and analyzed separately from cells that had not taken up mycobacteria (BCG-GFP^-^). Bar charts show mean and SEM and superimposed dots represent the results from three - six individual donors. Box graphs show mean - whiskers min to max values, and superimposed dots represent the results from three - six individual donors. Histogram overlays plots **(C, G)** are representative. Statistical significance was determined by two-way mixed model analysis of variance with Šídák’s multiple comparison tests. *p<0.05 and ***p<0.001, ns, not significant.

Inhibition of glycolysis significantly decreased the secretion of IL-1β, TNF-α and IL-10 by BCG-infected cells (**Figure 5**). Production of IL-6 was not altered by 2-DG. These results suggest that the glycolytic pathway is involved not only in the secretion of pro-inflammatory cytokines but also is necessary for optimal IL-10 production by CD1c^+^ mDCs.

### 3.5 Expression of the maturation markers CD40 and CCR7 in BCG-challenged CD1c^+^ mDCs is altered by inhibition of the glycolytic pathway

To further investigate the role of glycolysis in the effector function of CD1c^+^ mDCs in response to infection with BCG, we analyzed the cell surface expression levels of the costimulatory molecule CD40 and the chemokine receptor CCR7. The cells were once more pre-treated or not with 2-DG and challenged with BCG. Cell surface staining of the markers CD40 and CCR7 was analyzed by flow cytometry at 20h p.i. First, the analysis was performed considering all live cells in the cell population. As before, the percentage of CD40 positive cells and CD40 expression levels (measured as mean fluorescence intensity) were significantly increased in response to challenge with BCG. Inhibition of glycolysis with 2-DG significantly decreased the expression of CD40 (both percent positive cells and MFI) on BCG-challenged CD1c^+^ mDCs. Increased expression of CD40 was also observed with poly(I:C)/R848 stimulation compared to unstimulated cells and there was a trend towards decreased CD40 expression with 2-DG treatment, but this was not statistically significant (**Figures 5A, B**).

Most cells expressed CCR7 at baseline and the percentage of cells positive for CCR7 did not change significantly following challenge with BCG or treatment with 2-DG (**Figure 5E**). Surprisingly, surface expression of CCR7 was significantly increased after blocking glycolysis with 2-DG in infected cells. Expression levels of CCR7 were also significantly increased in poly(I:C)/R848 treated CD1c^+^ DCs with inhibition of glycolysis (**Figure 5F**).

As we used GFP-tagged BCG in our assays, we analyzed the surface levels of both CD40 and CCR7 maturation markers separately in the population of cells that successfully phagocytosed the mycobacteria, named here BCG-GFP^+^ cells, and those bystander cells which had been exposed to but had not engulfed any bacilli (BCG-GFP^-^ cells). The results for CD40 showed that for the bystander BCG-GFP^-^ CD1c^+^ mDCs, the surface levels of this marker were increased following challenge with BCG whereas inhibition of glycolysis for this cell population resulted in failure to up-regulate CD40. In contrast, in BCG-GFP^+^ CD1c^+^ mDCs, the expression levels of CD40 were up-regulated in response to challenge with BCG in both untreated and 2-DG treated cells indicative of differential effects of glycolysis on CD40 expression depending on whether or not the cells had phagocytosed the bacteria (**Figures 5C, D**).

Regarding the expression of CCR7 on the cell surface of CD1c^+^ mDCs following challenge with BCG, we observed that for the BCG-GFP^-^ cells, the levels of CCR7 were similar for both untreated and 2-DG-treated cells. However, for the BCG-GFP^+^ population, inhibition of glycolysis significantly increased CCR7 expression levels compared to untreated BCG-GFP^+^ CD1c^+^ mDCs (**Figures 5G, H**).

### 3.6 The migratory capacity of CD1c^+^ mDCs infected with BCG is not dependent on glycolysis

**Figure 6:**
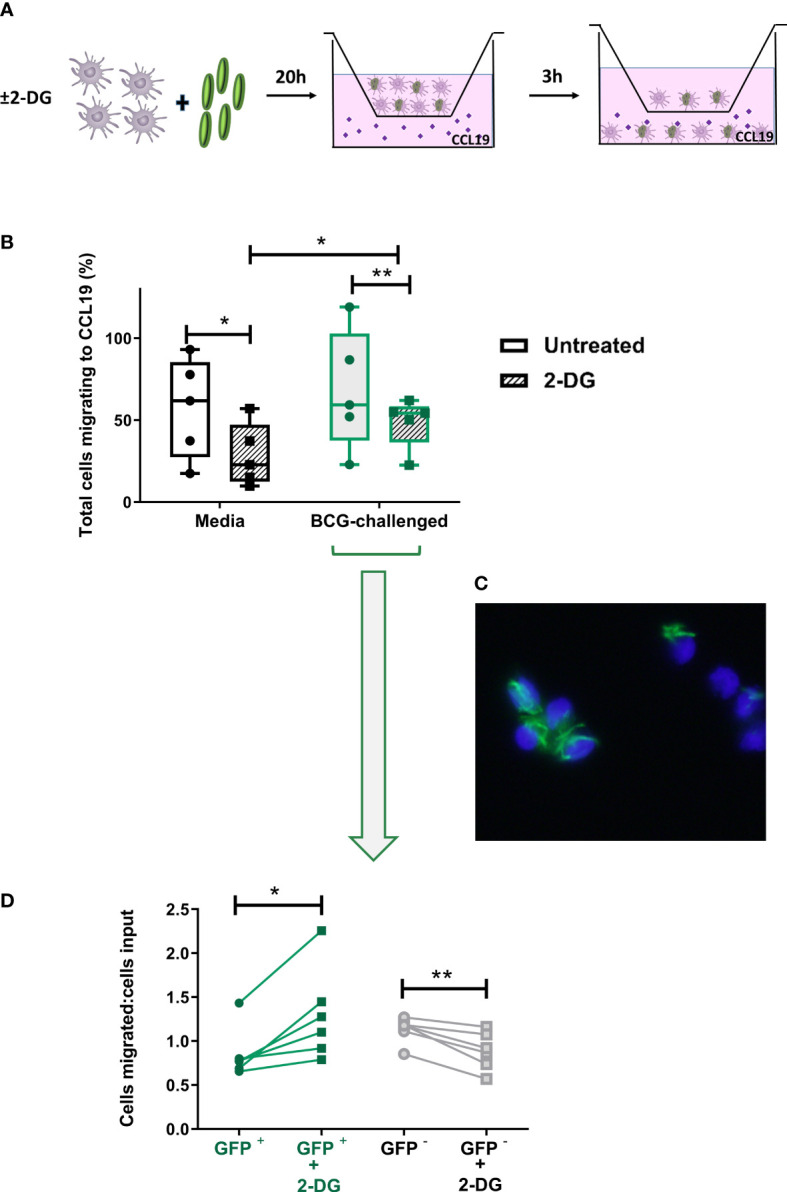
Inhibition of glycolysis has differential effects on CCR7-mediated migration of bystander and BCG-infected CD1c^+^ mDCs. **(A)** *In vitro* cell migration assay outline: CD1c^+^ mDCs were pre-treated or not with 1mM of 2-DG and challenged with BCG-GFP for 20h. They were then resuspended in fresh medium and allowed to migrate for 3h in a transwell system through a 5μm pore membrane toward a gradient of CCL19 chemokine (50ng/ml). The total number of cells migrating into the lower chamber after 3h was enumerated using a Cytell Imager. Results were calculated as the percentage of cells migrating to CCL19. **(B)** Graph shows data from five different donors as a percentage of initial number of cells added to the chamber (input). Box plots extend from the 25-75% interquartile range, the horizontal bar depicts the median and whiskers indicate the minimum to maximum values. Each dot represents the result from an individual donor. Data were analyzed using Two-Way mixed model ANOVA with Šídák’s multiple comparisons test. **(C)** Representative image of transmigrated CD1c^+^ mDCs obtained using an epifluorescent microscope, showing intracellular GFP^+^ bacilli. Cell nuclei were counterstained with Hoechst 33342. **(D)** Aliquots of the cells added to the top chamber, and transmigrating cells collected from the lower chamber after 3 hours, were placed on slides and fixed. For each condition 200 cells were counted using the 100X objective of an epifluorescent microscope and the numbers of BCG-infected (GFP ^+^) and uninfected cells (GFP ^-^) was calculated. Results are plotted as a ratio of the number of infected or uninfected cells added to the top chamber (input) divided by the number of infected or uninfected transmigrated cells with and without 2DG treatment. Graph shows data from six different donors. Statistical significance was determined using paired t-tests. *p<0.05 and **p<0.01.

Migration of infected mDCs to draining LNs is required to initiate an adaptive immune response to BCG (Bollampalli et al., 2015). Following our observation that treatment with 2-DG increased levels of CCR7 on the cell surface of BCG-infected CD1c^+^ mDCs, we set up a transwell migration assay to determine if inhibition of glycolysis influenced CCR7-dependent cell migration. CD1c^+^ mDCs, either unchallenged or challenged with BCG in the presence or not of 2-DG for 20h, were allowed to migrate for 3 hours through a membrane towards a gradient of CCL19, a ligand for CCR7 (**Figure 6A**). The numbers of transmigrated cells were counted by automated microscopy and then further analyzed by fluorescence microscopy to determine the proportion of GFP^-^ and GFP^+^ cells. Few DCs migrated through the membrane in both uninfected and BCG-challenged control samples where culture medium alone was added to the lower chamber of the transwell plate. The number of migrated cells was significantly higher when medium containing CCL19 was present in the lower chamber (**Supplementary Figure 5A**). Analysis of the total CD1c^+^ mDCs that migrated through the membrane showed that treatment with 2-DG significantly decreased the overall migration of immature CD1c^+^ mDCs towards CCR7 from 57.50% (+/-13.60%) to 28.45% (+/- 8.52%) of the original number of DCs added to the upper chamber (input), in line with previously published results obtained with murine BMDC migrating to a CCR7 ligand (CCL21) (Guak et al., 2018). Blocking glycolysis with 2-DG decreased the overall migration of BCG-challenged CD1c DCs from 68.09% (+/-16.34%) to 48.78% (+/-6.82%) of the input (**Figure 6B**).

As before, based on the presence of GFP-expressing BCG, it was possible to differentiate between uninfected bystander (GFP^-^) and BCG-infected (GFP^+)^ CD1c^+^ mDCs in the BCG-challenged cultures. Aliquots of the input and the transmigrated BCG-challenged cells from each donor - untreated (media) or treated with 2-DG - were mounted on slides and 200 mDCs per sample were visualised at 1000X magnification by fluorescent microscopy. We counted the numbers of input and migrated cells that were infected with BCG-GFP versus those bystander cells that were uninfected. The numbers of migrated BCG-infected and uninfected mDCs were normalised to the numbers of BCG-infected and uninfected mDCs respectively in the input. A significantly higher proportion of 2-DG-treated BCG-GFP^+^ mDCs migrated towards the chemokine stimulus relative to untreated BCG-GFP^+^ mDCs. Conversely, the proportion of BCG-GFP^-^ bystander cells in the same samples was significantly reduced in the presence of 2-DG (**Figure 6D**). The percentages of transmigrated GFP^+^ and GFP^-^ mDCs without normalisation are shown in **Supplementary Figure 5B**. Taken together, these data suggest that, although glycolysis potentiates the migration of immature and of mature bystander BCG-challenged CD1c DCs, glycolytic flux is not required for CCR7-mediated trafficking of BCG-infected CD1c^+^ mDCs.

## 4 Discussion

In this study, we characterized the metabolic profile and investigated the role of the glycolytic pathway in the response of CD1c^+^ mDCs the BCG vaccine. We found that CD1c^+^ mDC mature and acquire a more energetic phenotype upon challenge with BCG. Pharmacological inhibition of glycolysis with 2-DG decreased cytokine secretion and regulated the expression of both CD40 and CCR7 on BCG-challenged, compared to untreated, mDCs. In addition, inhibition of glycolysis had differential effects on infected and uninfected bystander mDCs in BCG-challenged cultures. To our knowledge, this is the first study investigating immunometabolism in human CD1c^+^ mDCs infected with mycobacteria.

A significant barrier to studying natural human DCs is their low frequency in blood and tissues which severely limits the number of assays that can be carried out on cells from any one donor. To optimize the purification procedure and assays and carry out the experiments presented here we processed blood from over 60 donors. Due to the low yield of viable DCs from buffy coats we isolated CD1c^+^ mDCs from freshly drawn blood collected from patients with Hereditary Hemochromatosis (HH). Our preliminary analysis showed no differences regarding expression of surface markers and endocytic capacity (assessed by Dextran-internalization assay) between mDCs from HH patients and healthy controls. We cannot rule out the possibility that there are differences between the response of HH and healthy control mDCs to BCG. However, a report investigating the characteristics of monocyte-derived DCs from HH patients showed no phenotype and functional differences at both the immature and mature stages in these cells (Phothirath et al., 2002). Despite the considerable challenges of working with these cells we believe it is important to carry out these studies using natural human DCs given that most of our knowledge of DC metabolism to date is based on *in vitro* studies of murine BMDCs or human monocyte-derived DCs which are ontologically and phenotypically distinct (Collin and Bigley, 2018) (Minarrieta et al., 2021).

Enhanced glycolysis is considered essential for the effector function and survival of activated immune cells, including DCs (Krawczyk et al., 2010; Everts et al., 2012). This increase in flux through glycolysis is associated with the high levels of protein and lipid synthesis that are necessary for DC functions such as cytokine secretion and increased cell surface expression of co-stimulatory molecules (Amiel et al., 2014). In line, we found that exposure of CD1c^+^ mDCs to BCG stimulated increased production of lactate and increased both the basal rate of glycolysis and the glycolytic capacity of human CD1c^+^ mDCs compared to immature mDCs. In contrast, the glycolytic reserve of BCG-challenged CD1c^+^ mDCs was similar to that of immature mDCs, suggesting that the highly glycolytic state induced in CD1c^+^ mDCs by BCG infection may exhaust their ability to further boost their rate of glycolysis. Although generally considered to play a pro-inflammatory role in DCs, a high glycolytic rate is also observed in tolerogenic mo-DCs, which are resistant to maturation and can inhibit T cell proliferation and cytokine production (Ferreira et al., 2015; Malinarich et al., 2015). BCG vaccination has been shown to induce both protective Th1/Th17 (Boer et al., 2015) and Treg responses in humans (Boer et al., 2015; Keefe et al., 2021). In agreement with a pro-inflammatory role for glycolysis in BCG-challenged DCs, we observed that treatment with 2-DG significantly decreased secretion of IL-1β and TNF-α by CD1c^+^ mDCs in response to BCG infection. Nonetheless, secretion of the anti-inflammatory cytokine IL-10 was also reduced after inhibition of glycolysis in human CD1c^+^ mDCs upon infection with BCG, like murine BMDCs stimulated with LPS (Krawczyk et al., 2010). Trafficking of BCG-infected CD11c^+^ DCs to draining LN is partially dependent on IL-1R signaling (Bollampalli et al., 2015) and both TNFα and IL-1β are crucial to host defence against Mtb infection (Mayer-Barber et al., 2010). IL-10 production, on the other hand, impairs the development of protective Th1/Th17 immune responses following BCG vaccination (Pitt et al., 2012).

Aerobic glycolysis is required to maintain the viability of iNOS-expressing murine DC subsets stimulated with LPS by providing ATP and metabolic intermediates in the face of sustained inhibition of mitochondrial respiration (Krawczyk et al., 2010; Everts et al., 2012; Everts et al., 2014; Thwe and Amiel, 2018). Our data, in contrast, show that the human CD1c^+^ mDCs population utilizes both pathways 20 hours after stimulation with BCG. This may reflect differences in the response to the stimulus used (LPS *versus* BCG), analogous to murine BMDCs stimulated with “weak” stimuli e.g. house dust mite - where an early increase in the rate of glycolysis is not sustained and does not lead to a concomitant decrease in OXPHOS - compared to “strong” stimuli (LPS, zymosan) which skew metabolism almost exclusively towards aerobic glycolysis (Guak et al., 2018). Basit and colleagues observed that TLR7/8 stimulation of human CD1c^+^ DCs with single-stranded RNA induces metabolic reprogramming by down-regulating OXPHOS *via* BNIP3-mediated mitophagy and inducing glycolysis to support maturation (Basit et al., 2018), indicating that this DC subset is capable of undergoing a Warburg-like switch to aerobic glycolysis under certain circumstances. Therefore, the more balanced response to BCG may be stimulus-dependent rather than a DC subset-specific response. Interestingly, TLR stimulation with poly(I:C)/R848 did not seem to inhibit OXPHOS either. Maintaining metabolic flexibility *in vivo* where maturing mDCs are competing with other immune cells for finite levels of nutrients could be an advantage (Amiel et al., 2012; Lawless et al., 2017). The bioenergetic requirements of mDCs responding to bacteria may also vary at different stages of maturation, in which case maintaining mitochondrial function could also be necessary to mount an effective immune response. Whether the changes we observed in ECAR and oxygen consumption are a true reflection of metabolic flexibility - whereby individual BCG-challenged cells increase their use of both pathways - or are the net result of heterogeneous metabolic responses of different mDCs is unclear and requires further investigation (discussed below).

BCG-challenged CD1c^+^ mDCs had increased expression of CD40 compared to unchallenged cells, which was significantly reduced by treatment with 2-DG. CD40 on antigen-presenting cells interacts with CD40L on CD4^+^ T cells to enhance IL-12 and IFN-γ production (Stuber et al., 1996), although Mtb impairs CD40-mediated induction of protective Th17 responses by DCs both *in vitro* and *in vitro* in mice (Sia et al., 2017). CD40 stimulation of BCG-infected murine BMDCs enhanced their capacity to produce IL-12 and activate a Th1 response (Demangel et al., 2001). In addition, treatment of antigen loaded murine BMDCs with a CD40 agonist before tracheal instillation increased protection against subsequent aerosol challenge with Mtb (Sia et al., 2017), highlighting the important protective role of this co-stimulatory molecule in immunity to Mtb.

Our data also indicated that CD40 expression is regulated differently in CD1c^+^ mDCs that successfully phagocytosed BCG-GFP (directly infected cells) compared to bystander cells which had been exposed to but had not taken up any bacilli. Both populations of cells upregulated CD40 levels on their cell surface after exposure to BCG but, whereas inhibition of glycolytic flux in the CD1c^+^ mDCs that had phagocytosed BCG resulted in sustained up-regulation of CD40, it was significantly decreased in GFP^-^ DCs treated with 2-DG. Bystander cells may have received signals from mycobacterial components released by infected cells (Beatty et al., 2000), through cell surface interaction with extracellular mycobacteria that were not subsequently phagocytosed and/or *via* cytokines or other inflammatory mediators released by infected cells. This may enable bystander cells to bypass pathogen-mediated inhibition of innate immune signaling in the directly infected cell (Holmgren et al., 2017). Indeed, BCG retains immunomodulatory factors like Hip1 that negatively impact DC responses, including CD40 expression, leading to impaired control of Mtb after challenge (Bizzell et al., 2018). Bystander effects have been observed previously in murine DCs infected with BCG: secretion of IL-12p40 was impaired in directly infected splenic CD11c^+^ DCs from mice expressing a YFP-tagged IL-12p40 gene and bystander DCs were responsible for IL-12p40 secretion (Rothfuchs et al., 2009). Moreover, migratory lung DCs infected with Mtb transfer antigen to bystander DCs in the mediastinal LN to stimulate CD4^+^ T cell proliferation (Samstein et al., 2013; Srivastava and Ernst, 2014) (Srivastava et al., 2016).

Migration of CD11b^+^ cDCs from the skin to draining LN is required for induction of adaptive immunity after intradermal injection of BCG in the mouse footpad model (Bollampalli et al., 2015). DC migration from sites of Mtb infection in the lung to the mediastinal LN is also required to initiate an adaptive immune response in murine models of TB (Khader et al., 2006; Wolf et al., 2007; Wolf et al., 2008) and is highly dependent on CCR7 signaling (Olmos et al., 2010). Inhibition of glycolysis by glucose starvation or treatment with 2-DG has previously been shown to constrain the migration of murine BM and splenic DCs *in vitro* to the CCR7 ligand CCL21 by inhibiting oligomerization of CCR7 without altering expression levels of CCR7 (Guak et al., 2018). Guak and colleagues also found that inhibition of glycolysis impaired LPS- or house dust mite-stimulated DCs migrating to draining LN *in vivo* in sensitized mice (Guak et al., 2018). CCR7 signaling alone can also trigger metabolic reprogramming by increasing HIF1-α-mediated expression of glycolytic genes in murine BMDCs (Liu et al., 2019). In line, we found that expression of CCR7 on immature human CD1c^+^ mDCs was unchanged by treatment with 2-DG although their migration towards CCL19 was inhibited. Consequently, it was surprising that glycolysis blockade significantly increased cell surface expression of CCR7 on BCG-challenged and poly(I:C)/R848-treated CD1c^+^ mDCs although it did significantly inhibit migration of BCG-challenged CD1c^+^ mDCs.

Analogous to CD40 expression, we observed divergent effects of glycolysis inhibition on CCR7-mediated migration of bystander and BCG-infected cells. While CCR7-mediated migration of bystander cells was reduced by 2-DG treatment, BCG-infected cells in the same population displayed higher migratory capacity toward the chemokine stimulus. Since a fixed number of cells were counted and the data was proportional, an increase in infected cells was inevitably accompanied by a decrease in uninfected cells. Therefore 2-DG may have inhibited the migration of bystander cells and/or enhanced the migration of infected cells. Nevertheless, our data clearly show that migration of BCG-infected DCs does not depend on glycolysis - in contrast to that of bystander DCs - and that CCR7 levels are increased on infected cells when glycolytic flux is inhibited. Although immune cell migration is generally thought to be dependent on aerobic glycolysis, there are several reports showing that cell motility can be inhibited by glycolysis or improved by inhibition of glycolytic flux. For example, reduced cell migration has been observed in migratory DCs from UV-irradiated mice, despite higher lactate production and increased glycolytic rates compared to DCs from non-irradiated mice (McGonigle et al., 2017). In addition, murine CD8^+^ T cells treated with 2-DG *in vitro* also upregulate CCR7 mRNA. Adoptive transfer of these 2-DG-treated CD8^+^ T cells resulted in their preferential migration to the LN where they adopted a memory phenotype (Sukumar et al., 2013). Treatment with 2-DG is likely to have skewed mDC metabolism towards OXPHOS therefore it is tempting to speculate that BCG-infected DCs require OXPHOS for efficient migration, in contrast to bystander DCs and, at the very least, can migrate when glycolysis is inhibited. In fact, BMDCs infected with influenza virus require mitochondrial respiration for optimal motility despite being predominantly dependent on glycolysis for ATP production (Rezinciuc et al., 2020). Changes in mitochondrial dynamics have also been observed to influence CCR7 expression and migration of immature murine BMDCs (Ryu et al., 2015). Since delayed migration of Mtb-infected migratory DCs impairs the development of adaptive immunity (Wolf et al., 2007; Lai et al., 2018), identifying the mechanisms that regulate CCR7-dependent migration in infectious disease may help to inform the design of new therapeutics and improved vaccines and requires further investigation.

The increase in basal OCR we observed following the addition of glucose to BCG-infected mDCs suggests an increased ability of BCG-challenged cultures to utilize glucose for mitochondrial respiration compared to control mDCs (Pike Winer and Wu, 2014). Conversely, it should also be acknowledged that 2-DG treatment may have inhibited glucose-dependent OXPHOS by reducing pyruvate availability for the TCA cycle. In addition to generating lactate as an end product, engagement of the glycolytic pathway by activated DCs can also channel glucose through both the TCA cycle and the ETC (Pike Winer and Wu, 2014) or *via* a truncated TCA cycle to generate citrate for fatty acid synthesis (FAS) (Moller et al., 2022) enabling expansion of the Golgi and ER membranes required for cytokine secretion and the formation of lipid mediators (Everts et al., 2014) as well as fueling fatty acid oxidation. Increased FAS has been observed in murine DCs infected with BCG and, while not required to control the growth of mycobacteria *in vitro* (Stuve et al., 2018), it may impact other aspects of DC function important for protective immunity. The observed increase in non-glycolytic ECAR in BCG-challenged cultures might also reflect increased oxygen consumption, as CO_2_ produced by the TCA cycle can be a source of extracellular acidification *via* the formation of carbonic acid (Mookerjee et al., 2015). However, glycogenolysis can also contribute to ECAR and DCs are known to have intracellular stores of glycogen which can be utilized to fuel glycolysis (Thwe et al., 2017) and which could have contributed to ECAR in the absence of glucose in the medium. In the present study we focused primarily on the role of glycolysis in mDC maturation, but it will be important in the future to assess the contribution of OXPHOS to the ability of these cells to stimulate an effective immune response to mycobacteria.

Taken together, our data indicate that maturation and migration of CD1c^+^ mDCs in response to BCG does not rely solely on the glycolytic pathway once the cells have phagocytosed the mycobacteria. Importantly, our data indicate that significant differences can occur between BCG-infected versus uninfected bystander DCs in terms of activation marker expression and cell migration, as well as in the regulation of these processes by glycolysis. Most of the limitations of this study arise from the logistical difficulties of performing functional assays with a rare population of primary cells. The Seahorse metabolic flux assays carried out to measure the bioenergetic profile of the DCs were performed, by necessity, on the entire BCG-challenged population of DCs. Therefore, it is possible that the differences we observed in expression of maturation markers and migratory capacity could also reflect metabolic heterogeneity of infected and uninfected populations of DCs, as alluded to above. Due to restricted cell numbers, it was not possible to sort sufficient BCG^+^ and BCG^-^ cells to perform separate metabolic flux measurements in our study. Additionally, it has recently become clear that CD1c ^+^ mDC comprise a rather heterogeneous population of cells and that the recently characterized cDC3 subset also expresses CD1c. Based on our sorting strategy which excludes Lin^+^ cells, and therefore CD14^+^ DCs, it is likely that our population of CD1c^+^ mDCs are representative of cDC2 (Segura, 2022) but this will require additional investigation. Further analysis - perhaps using flow cytometry based single cell methods such as SCENITH (Arguello et al., 2020) and/or ssRNAseq to compare metabolism/metabolic gene expression and cell surface markers in the uninfected and infected cell populations - will be required to determine if CD1c^+^ mDCs metabolism differs between bystander and BCG-infected cells. Such methods might also allow for comparison of the metabolism of healthy control and HH mDCs in PBMCs, thus avoiding the necessity to isolate pure subset populations from large volumes of blood using stringent sorting protocols. This is important given the association of iron with cellular metabolism (Zhang et al., 2022). Another limitation is that we were unable to carry out extracellular flux analysis of DCs infected with live BCG due to biosafety restrictions in our institution. However, the observed increased in lactate secretion by CD1c^+^ mDCs infected with live BCG indicate that glycolysis is indeed increased by live as well as killed BCG infection. Similar to our results in DCs with killed BCG, live BCG was shown to increase both OCR and ECAR of human monocytes (Arts et al., 2016) and monocyte-derived macrophages (Cumming et al., 2018). However, it will be important going forward to determine whether there are differences between the effects of live and killed BCG on DC metabolism.

In conclusion, our results identify metabolic pathways in CD1c^+^ mDCs as potential targets to promote the protective immunity given by the BCG vaccine in tuberculosis infection. However, the disparate effects of glycolysis on bystander and infected CD1c^+^ mDCs function following exposure to mycobacteria highlight the complexity of this host response. Our data also suggest that there may be differences in the immunometabolic response of host cells to vaccines consisting of whole bacteria versus those composed of bacterial-derived antigens. In addition, this work raises several important questions outside the scope of the current study: i) what is the role of mitochondrial metabolism in DC maturation and migration? ii) what is the basis of the differences in metabolic requirements between bystander and BCG-infected DCs? iii) Is DC glycolysis required to stimulate optimal T cell responses to BCG and thus adaptive immunity against Mtb? Further investigation, including testing of these pathways in animal models of infection, will be needed to evaluate the feasibility of manipulating mDC metabolism to aid rational vaccine design.

## Data availability statement

The raw data supporting the conclusions of this article will be made available by the authors, without undue reservation.

## Ethics statement

The studies involving human participants were reviewed and approved by St James Hospital and Tallaght University Hospital Joint Research Ethics Committee. The patients/participants provided their written informed consent to participate in this study.

## Author contributions

DT and MO’S designed and planned the study. DT, MO’S and KG performed experiments, DT and MO’S analysed the data. MO’S and JK obtained funding. DT wrote the first draft of the manuscript, MO’S and DT wrote sections of the manuscript. All authors contributed to manuscript revision, read, and approved the submitted version.
