# Supplementary material for: Glucose metabolism and its role in the maturation and migration of human CD1c+ dendritic cells following exposure to BCG
Source: Front Cell Infect Microbiol. 2023 Jul 5;13:1113744. doi: 10.3389/fcimb.2023.1113744 (PMC10354370; doi:10.3389/fcimb.2023.1113744)
Supplement: Supplementary file 1 [file DataSheet_1.pdf]

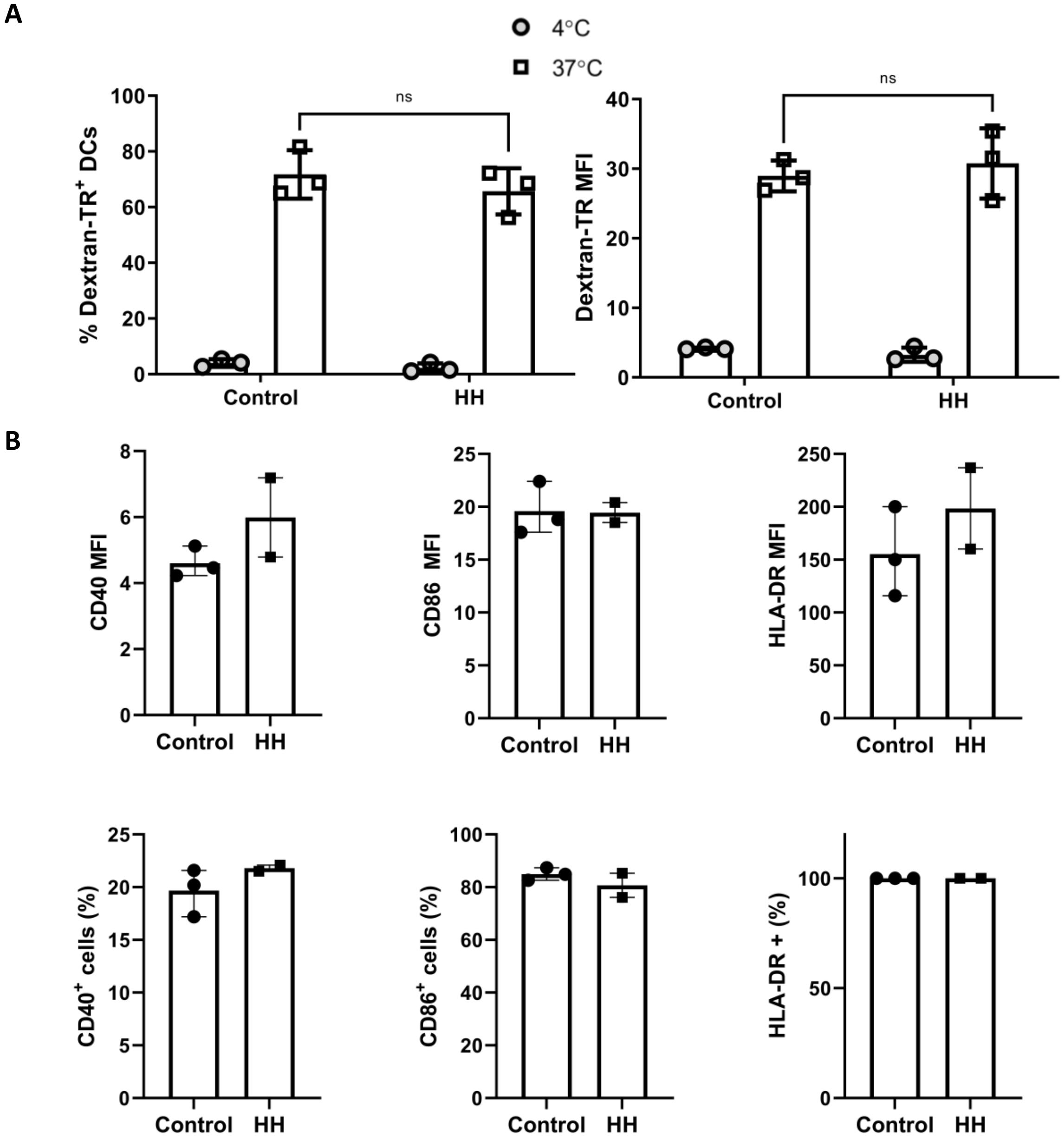

Supplementary Figure 1. Comparison of endocytic function and activation status of mCD1c+ DC in PBMCs isolated from healthy controls and individuals with hereditary Haemochromatosis. (A) PBMCs were isolated from the peripheral blood of healthy controls and individuals with hereditary haemochromatosis (HH) and incubated with dextran conjugated to Texas red for 24h at either 4°C or 37°C. Cells were then washed, stained with antibodies to cell surface markers and subjected to analysis by flow cytometry to identify mCD1c+ DCs. Dextran uptake is represented as percent Texas red (TR) positive mCD1c+ DC and Texas red MFI. Data were analysed using unpaired t tests. Graphs show data from three independent donors. (B) PBMCs were incubated with antibodies to cell surface markers and analyzed by flow cytometry to identify mCD1c+ DC. Graphs show expression of CD40, CD86 and HLA-DR by the mCD1c+ DC (from two (HH) to three (healthy control) donors).

A

B

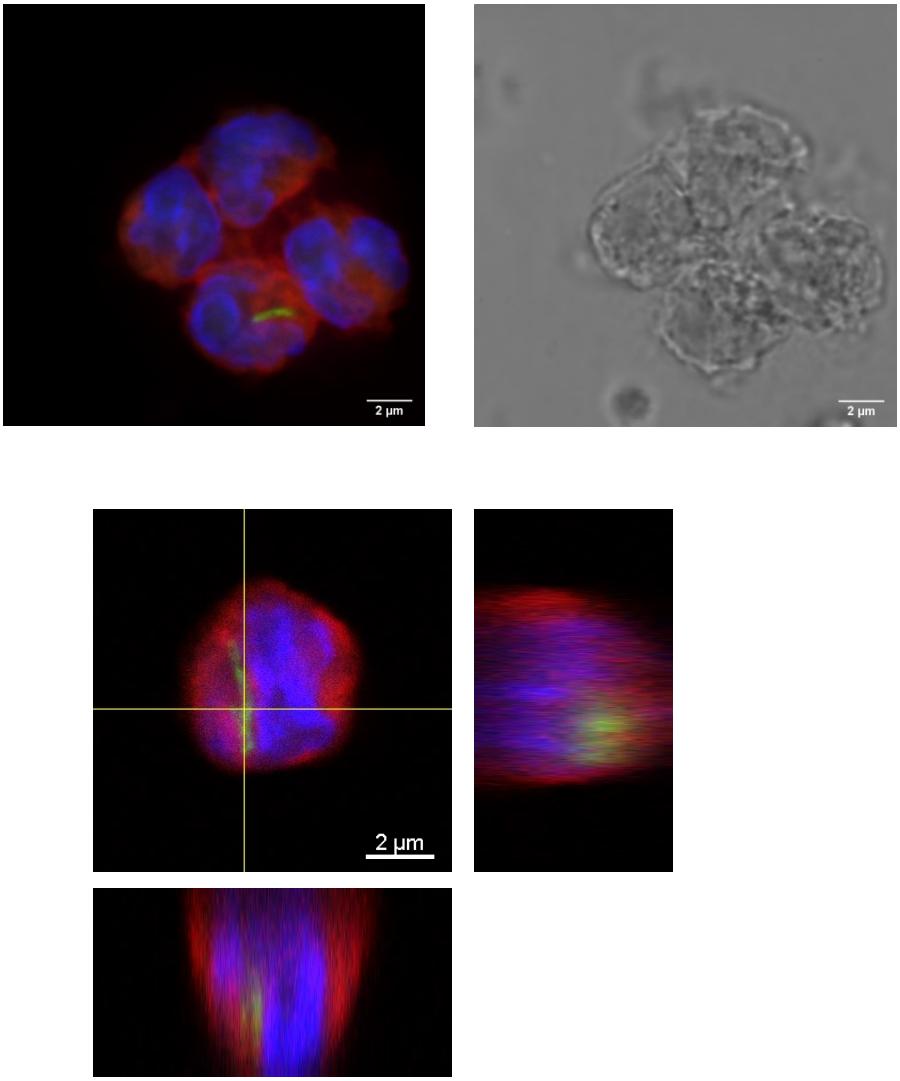

Supplementary Figure 2. **Confocal microscopic analysis of human CD1c^{+} mDCs following challenge with BCG.** Sorted human CD1c^{+} mDCs were incubated for 20h with BCG-GFP (MOI 10), washed, mounted on microscope slides and analysed by confocal microscopy. A) Representative image of CD1c^{+} mDCs obtained using a ×63 oil immersion objective showing overlay of nuclei stained with Hoescht 33342 (blue), BCG-GFP in green and cytoplasm (red) on the left and bright field image on the right. B) For 3D acquisition, z-stacks were obtained using a ×63 oil immersion objective displaying nuclei in blue, BCG-GFP in green and cytoplasm (red). Scale bar = 2 microns. Images shown are representative of five individual donors.

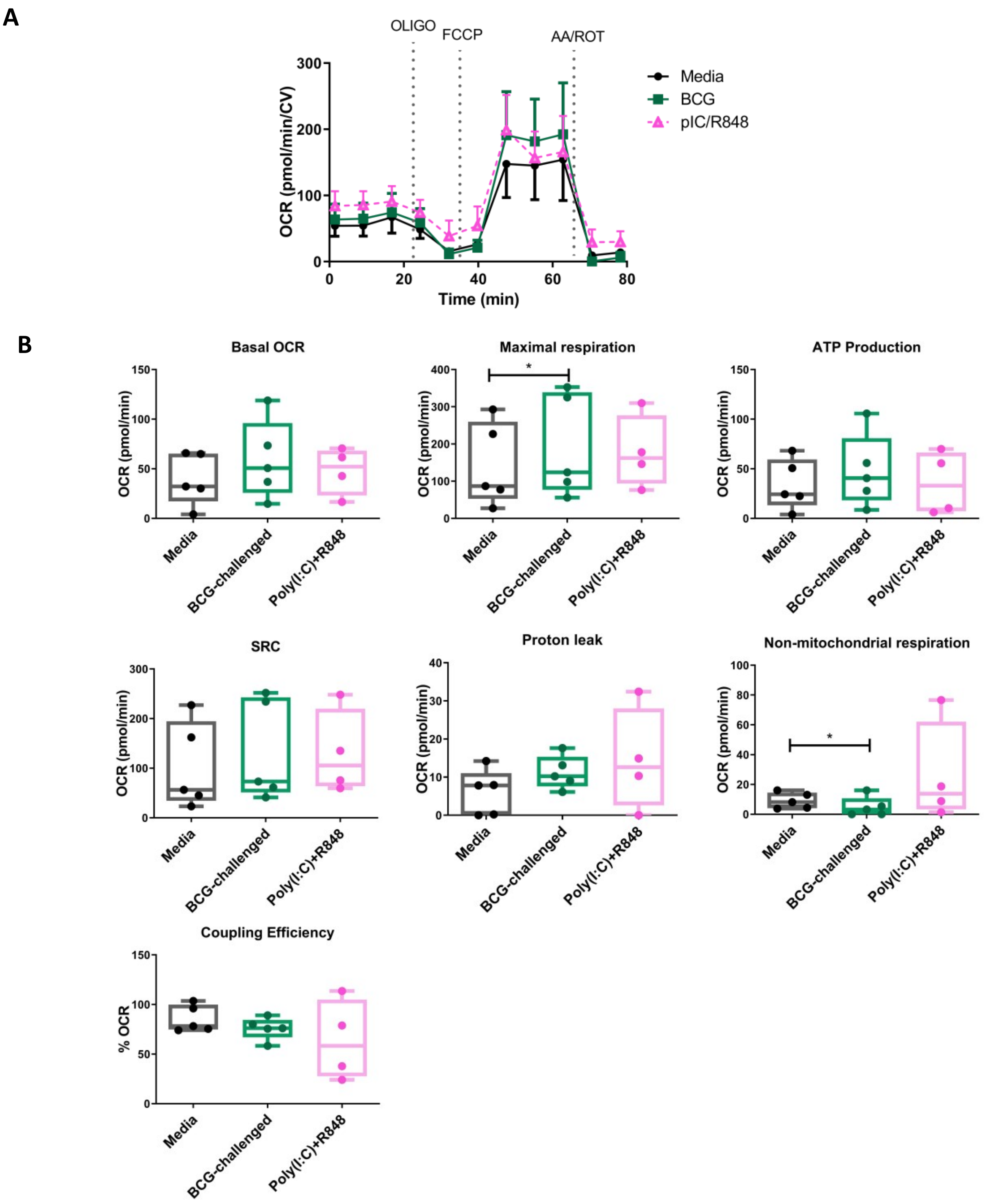

Supplementary Figure 3. **Analysis of mitochondrial function in CD1c^{+} mDCs following challenge with BCG.** A) OCR changes as determined by Seahorse metabolic profiling. CD1c^{+} mDCs were challenged with BCG-GFP or treated with poly(I:C)+R848 for 20h. OCR was measured using the Mito Stress test. B) Basal respiration (last measurement before injection of oligomycin minus non-mitochondrial respiration rate), maximal respiration (maximum rate measurement after FCCP injection minus non-mitochondrial respiration), ATP production (last measurement before injection of oligomycin minus minimum rate measurement after oligomycin injection), spare respiratory capacity (maximal respiration minus basal respiration), coupling efficiency (ATP production rate minus basal respiration), proton leak (minimum rate measurement after oligomycin injection minus non-mitochondrial respiration). Box plots extend from the 25-75% interquartile range, the horizontal bar depicts the median and whiskers indicate the minimum to maximum values. Superimposed dots represent the results from six (BCG) and four (poly(I:C)+R848) independent donors. * $p < 0.05$ (Repeated measures ANOVA (mixed model) with Dunnet's multiple comparisons test).

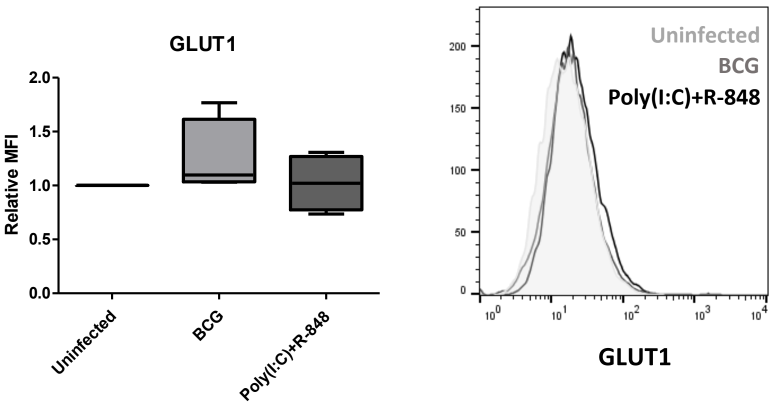

Supplementary Figure 4. GLUT1 surface staining measured by flow cytometry in CD1c mDCs following infection with BCG or stimulation with TLR ligands. Box graph show data from four independent donors (Whiskers: Min to Max values). Histogram overlays plot is representative.

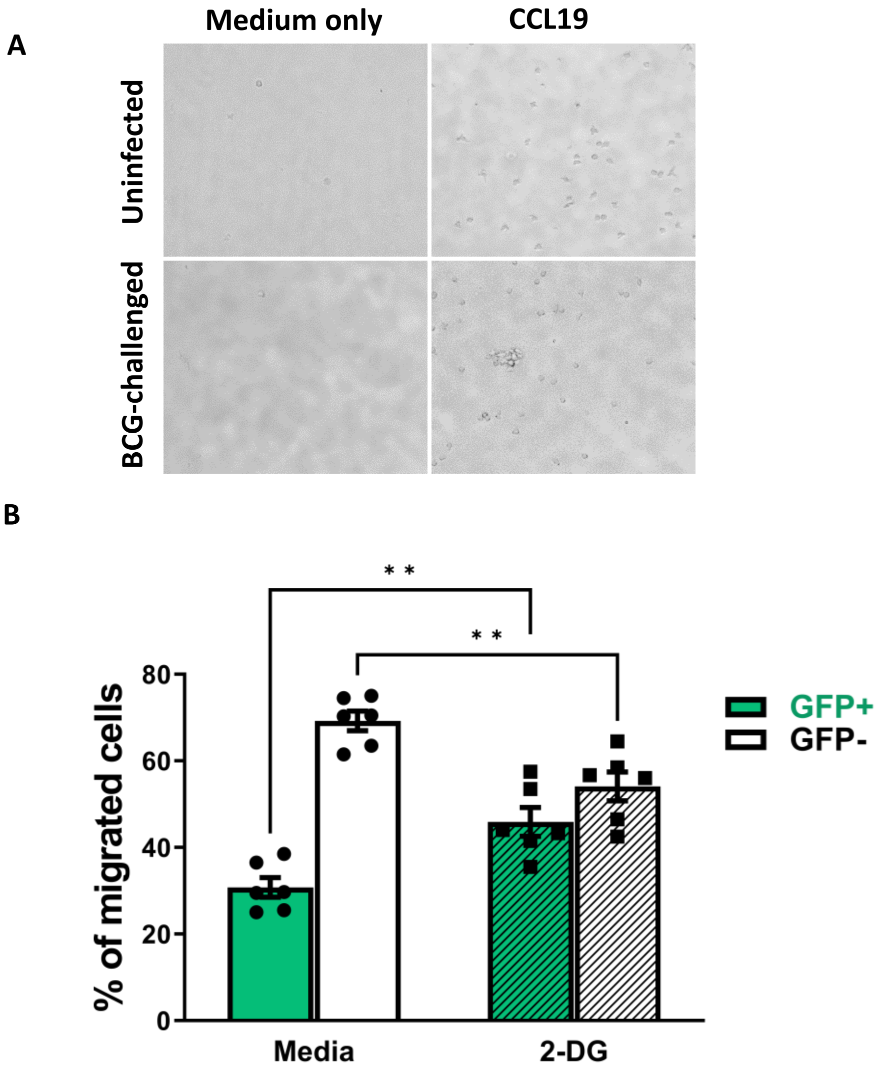

Supplementary Figure 5. (A) Bright field images of CD1c^{+} mDCs on the bottom chamber of the transwell plate used in the migration assay described in Materials and Methods. Cells were infected or not with BCG and then allowed to migrate toward CCL19 (50ng/ml) or culture medium only. Images were taken after 3 hours under a 10X objective using an Olympus IX51 microscope. (B) The percentages of transmigrated GFP^{+} and GFP^{-} cells following challenge with BCG-GFP in the absence (Media) or presence of 1mM 2-DG (n=6) was determined by fluorescence microscopy using a 100X oil immersion objective. ** p< 0.01 (Two-way ANOVA with Šidáks multiple comparisons test).
